# Water-Soluble Silicon Quantum Dots with Quasi-Blue Emission

**DOI:** 10.1186/s11671-015-1012-2

**Published:** 2015-07-25

**Authors:** Yun Wang, Hao Wang, Jun Guo, Jiang Wu, Li J. Gao, Ying H. Sun, J. Zhao, Gui F. Zou

**Affiliations:** College of Physics, Optoelectronics and Energy & Collaborative Innovation Center of Suzhou Nano Science and Technology, Soochow University, Suzhou, 215006 People’s Republic of China; Testing and Analysis Center, Soochow University, Suzhou, People’s Republic of China; State Key Laboratory of Electronic Thin Film and Integrated Devices, University of Electronic Science and Technology of China, Chengdu, 610054 People’s Republic of China

**Keywords:** Water-soluble, Quasi-blue emission, Silicon quantum dots

## Abstract

In this study, water-soluble silicon quantum dots have quasi-blue emission at 390 nm by being capped with 1-vinylimidazole in resese micelles. As-obtained silicon quantum dots have a diameter of 2~5 nm and high crystallinity. The quasi-blue emission of the silicon quantum dots is likely attributed to the polarity of the capping ligands. Moreover, the silicon quantum dots are water-soluble and have photoluminescence nanosecond decay time, suggesting their potential application in biological field.

## Background

In the recent years, extensive research effects have been dedicated to silicon quantum dots (Si QDs) due to their potential applications in a wide range of areas, such as photovoltaic, [1–3] light-emitting devices [4, 5], and biological fields [6–8]. Si QDs show the strong quantum effects and the increasing photoluminescence (PL) intensity by radiative recombination and direct band gap transitions to reduce phonon-assisted indirect band gap transitions.

Over the last decades, researchers have made great efforts to obtain Si QDs with different emissive light. In general, their emission peak can be tuned by changing the particles size or their capping ligands [9–14]. The tunable emission wavelength of Si QDs in the visible range suggests that these materials are promising candidates for bio-applications. As reported, Si QDs with oxide surface passivation typically exhibit dipole-forbidden yellow-red emission [15, 16]. However, they have a long radiative lifetime (10^−6^–10^−3^ s), which heavily limits their application in biological imaging. Fortunately, once passivated by a hydrogen or carbon atoms, the Si QDs will show blue emission and have electric-dipole-allowed direct band gap transitions which lead to a shorter radiative lifetime (10^−9^–10^−8^ s) [15, 17] and are suitable for biological imaging. To date, many blue PL Si QDs were designed and synthesized by different methods [12, 18, 19]. However, only few of them were water-soluble [20], which still cannot nearly satisfy the growing need for biological study. Therefore, the requirement of blue PL stable and water-soluble Si QDs remains.

Herein, we report a kind of water-soluble and stable Si QDs by the selection of 1-vinylimidazole to coat silicon quantum dots in reverse micelles. The As-synthesized Si QDs manifest a quasi-blue light emission peak at 390 nm and red-shifted around 50 nm compared to 1-heptene-capped ones. Those obtained Si QDs were mono-dispersed with a size distribution below 5 nm and high crystallinity. All these characterizations indicated that the water-soluble Si QDs were feasible for further applications in biological areas.

## Methods

### Synthesis and Purification of Alkyl-Si QDs

Si QDs were synthesized by the solution-phase reduction of SiCl_4_ in reverse micelles as reported. All reagents and manipulations were carried out in an argon atmosphere in a glove box to prevent oxidation of Si QDs. Si QDs were formed by adding 5.2 ml of the reducing agent (dropwise), 1 M of lithium aluminum hydride (LiAlH_4_) in tetrahydrofuran (THF) (strong reducing agent), to 50 ml of toluene containing 1 g of tetraoctylammonium bromide (TOAB) and 300 μl of SiCl_4_ in a 100-ml round-bottomed flask (the solution has been stirred for 1 h). After finishing adding the reducing agent, the solution was left to react for 3 h. Then the solution was quenched with 50 ml of anhydrous methanol. After stirring for several minutes, 100 μl of 0.1 M hexachloroplatinic acid (H_2_PtCl_6_) in isopropyl alcohol was added in the mixture. The surface-capping process was performed by adding 0.7 ml of 1-vinylimidazole into the solution. The solution was left to react for another 3 h. After that, all the solvents were removed from the mixture by rotary evaporation. The resulting gray white powder was redispersed in 20 ml of water for further purification and/or solvent exchange. The solution was filtered through 0.45 and 0.22 μm membrane filter twice to remove the excessive TOAB, and then a clear solution of Si QDs in water was obtained. To 1-heptene-capped Si QDs, they were distributed in hexane and the received solution was also washed with 20 ml of n-methyl formamide and finally with distilled water.

### Characterization

UV-vis absorption spectra were collected using a Shimadzu spectrophotometer (mode UV2450). PL and lifetime spectra were recorded by a Horiba spectrofluorometer (Fluoromax-4) using an excitation wavelength of 290 nm. Transmission electron microscope (TEM) studies were performed using a FEI Tecnai G2 F20 microscope operating at 200 kV. The particles size distribution was measured by Malvern Nano ZS.

## Results and Discussion

**Scheme 1:**
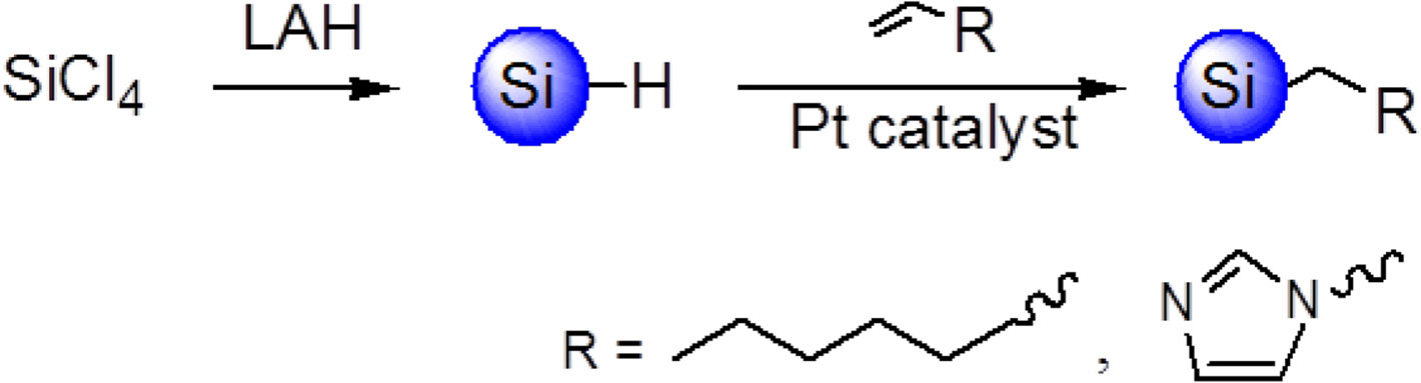
Synthesis route of Si QDs capped with 1-heptene and 1-vinylimidazole, respectively

**Fig. 1:**
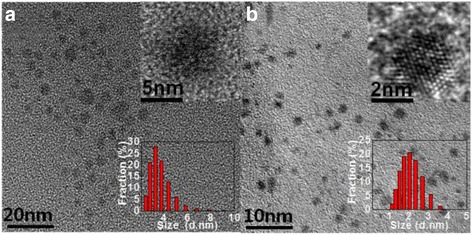
TEM and HRTEM images of **a** 1-heptene-capped Si QDs and **b** 1-vinylimidazole-capped Si QDs. Insets (*upper*) show a single alkyl-capped Si QD with clear lattice fringes. Insets (*lower*) are size distribution histograms of 1-heptene-capped Si QDs and 1-vinylimidazole-capped Si QDs

The two integrated reaction processes are presented as Scheme 1. As we know, 1-vinylimidazole is more water-soluble than 1-heptene, leading to 1-vinylimidazole-capped Si QDs hydrophilic and 1-heptene-capped Si QDs hydrophobic. As depicted in the TEM (Fig. 1), the As-synthesized-alkyl-capped Si QDs are almost spherical and the distribution of both 1-heptene- and 1-vinylimidazole-capped Si QDs are relatively mono-dispersed, showing the alkylation occurrence on the Si quantum dots surface to result in non-aggregation. From the high-resolution transmission electron microscope (HRTEM) images (Fig. 1 insets (upper)), a single particle possesses the clear lattice fringes (0.21 nm) of Si quantum dots. It is consistent with the (211) plane in diamond crystalline silicon. It suggests that the Si QDs with high quality are synthesized. From the dynamic light scattering (DLS) histogram of the As-prepared Si QDs (Fig. 1 insets (lower)), the size distribution of Si QDs manifests alkyl-capped silicon QDs in a solution having an average size of 3.2 ± 0.4 nm and 1.5 ± 0.3 nm, respectively, which are identical with the estimated results of TEM characterization.

**Fig. 2:**
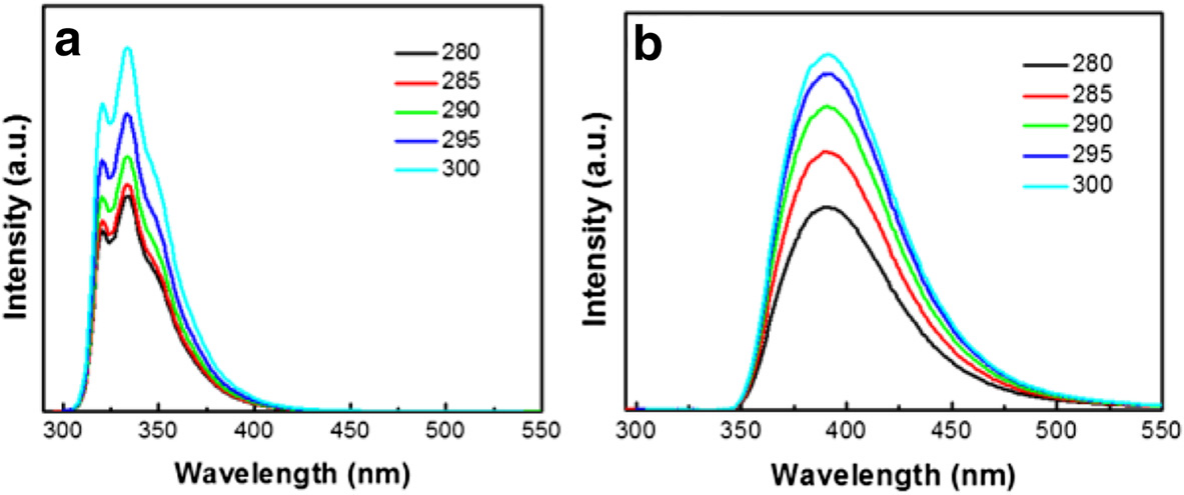
PL spectra of **a** 1-heptene-capped Si QDs in hexane and **b** 1-vinylimidazole-capped Si QDs in water excited with different wavelengths from 280 to 300 nm with a 5-nm increment

It is well-known that light emission of Si nanostructures can be greatly affected by the surface ligand modification [21]. Even tiny differences in the styles or structures of ligands modification may lead to apparent changes in Si QDs PL. Moreover, there is a theoretical demonstration that 1–2 nm Si QDs with a hydrogen or carbon surface termination have direct band gap optical transitions. This will lead to PL staying in the UV/blue region of the electronic spectrum [9]. As shown in Fig. 2, an intense emission peak at 335 nm originates from the pristine silicon character. Nevertheless, when 1-heptene-capped Si QDs are excited by different wavelengths, two shoulder peaks at 321 nm and 350 nm may be caused by the inhomogeneous size distribution. When the Si surface is modified by 1-vinylimidazole, the Si QDs exhibit an intense and regular emission peak at 390 nm, which is red-shifted about 55 nm in comparison with the 1-heptene-capped ones. The difference of our Si QDs’ emission peaks derives from the capping agents. As previously reported [22], the emission peak position is closely related with the capping ligands polarity. The emission peak of Si QDs capped with polar ligands is red-shifted compared with nonpolar capping ligands. This is consistent with our results. To one’s interest, there are no shoulder peaks along with the main Si emission peak. It can be indirectly told that the particles size seems to be more uniform, which is consistent with the above TEM characterizations.

**Fig. 3:**
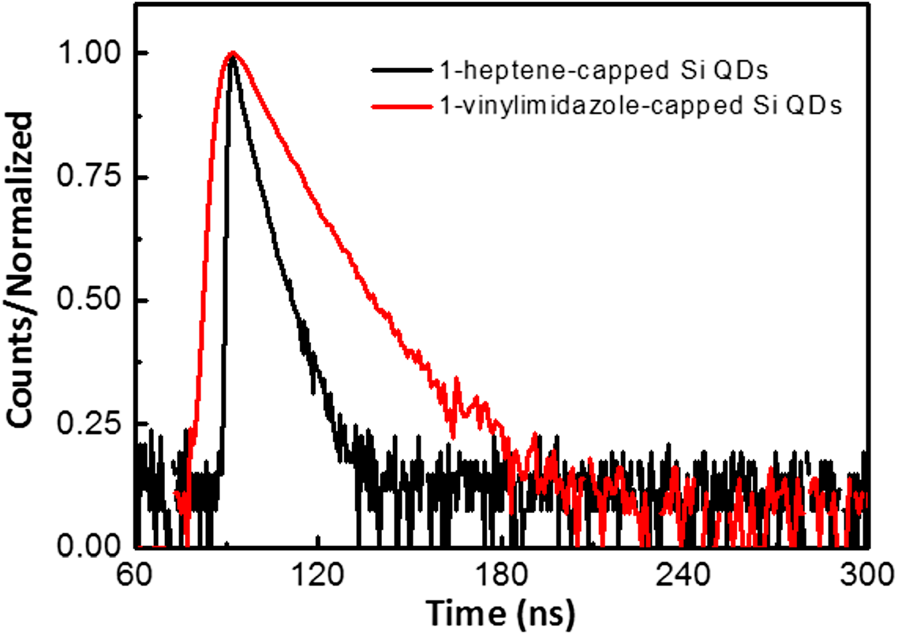
Fluorescence decay as a function of time (ns) for 1-heptene-capped silicon QDs (*black line*) in hexane measured at an emission wavelength of 335 nm and 1-vinylimidazole-capped Si QDs (*red line*) at an emission wavelength of 390 nm. A laser source of 300 nm was used

As reported in previous literature, time-resolved spectroscopy reveals the lifetime of excited states before radiative recombination, and this provides insights into the recombination pathways in the material [23]. As shown in Fig. 3, the PL average decay of 1-heptene-capped Si QDs in hexane and 1-vinylimidazole-capped Si QDs in water are estimated to be 3.84 and 1.93 ns by two-exponential fit of both PL decay, respectively. The rapid rates of recombination measured also indicate that the observed emission results from dipole-allowed recombination across the direct band gap transition in silicon quantum dots with a carbon surface termination [20] Meanwhile, the PL lifetimes agree well with others’ previous reports of PL decay measurements and with other reports of colloidal Si QDs with direct band gap emission [9, 17, 20].

**Fig. 4:**
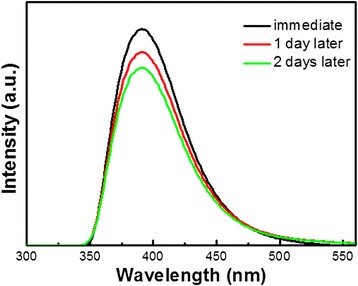
PL stability of 1-vinylimidazole-capped Si QDs

As well known, the stability of Si QDs is very important to the following applications. In Fig. 4, PL intensity of Si QDs is studied at different times. In our experiments, PL spectra of 1-vinylimidazole-capped Si QDs are measured upon preparation, 1 day, and 2 days later, respectively. It can be seen that the PL intensity of 1-vinylimidazole-capped Si QDs decreased by 8.3 % after being synthesized 1 day later. While the PL intensity only decreases by 1.8 % after 2 days. It can tell that 1-vinylimidazole-capped Si QDs remain to be stable. Based on our experience, the alkenyl-imidazole or analog ligands may modify the Si QDs as well. This provides a platform to find the new phenomena for the modified Si QDs.

Some researches about PL sensitivity to surrounding changes have been reported before [24, 25]. For example, as reported in a previous paper, the authors investigated nanosecond photoluminescence processes in colloidal core/shell CdSe/ZnS nanoparticles dissolved in water and found strong sensitivity of luminescence to the solvent state. Identically, the intensity of PL of our Si QDs are very strong, it is believable that they are sensitive to various changes in solvent, such as chemical and physical changes, via ligand reconstruction.

## Conclusions

In summary, Si QDs with a size distribution within 2~5 nm are synthesized by a micro-emulsion colloidal solution. Using a capping ligand of 1-vinylimidazole, we can gain a water-soluble and quasi-blue light emissive (at 390 nm) Si QDs. Based on the test of time-resolved PL, it can be seen that the decay of 1-vinylimidazole-capped Si QDs are around 1.93 ns. The synthetic method may be extended to other similar capping ligands to Si QDs.

## Abbreviations

DLS: dynamic light scattering
H_2_PtCl_6_: hexachloroplatinic acid
HRTEM: high-resolution transmission electron microscope
LiAlH_4_: lithium aluminum hydride
PL: photoluminescence
Si QDs: silicon quantum dots
TEM: transmission electron microscope
THF: tetrahydrofuran
TOAB: tetraoctylammonium bromide

## References

[1] Conibeer G, Green M, Cho EC, König D, Cho YH, Fangsuwannarak T (2008). Silicon quantum dot nanostructures for tandem photovoltaic cells. Thin Solid Films.

[2] Conibeer G, Green MA, König D, Perez-Wurfl I, Huang S, Hao X (2011). Silicon quantum dot based solar cells: addressing the issues of doping, voltage and current transport. Prog Photovolt: Res Appl.

[3] Cho EC, Park S, Hao X, Song D, Conibeer G, Park SC (2008). Silicon quantum dot/crystalline silicon solar cells. Nanotechnology.

[4] Ghosh B, Masuda Y, Wakayama Y, Imanaka Y, Inoue J, Hashi K (2014). Hybrid white light emitting diode based on silicon nanocrystals. Adv Funct Mater.

[5] Pillai S, Catchpole KR, Trupke T, Zhang G, Zhao J, Green MA (2006). Enhanced emission from Si-based light-emitting diodes using surface plasmons. Appl Phys Lett.

[6] Li ZF, Ruckenstein E (2004). Water-soluble poly(acrylic acid) grafted luminescent silicon nanoparticles and their use as fluorescent biological staining labels. Nano Lett.

[7] Erogbogbo F, Yong KT, Roy I, Xu G, Prasad PN, Swihart MT (2008). Biocompatible luminescent silicon quantum dots for imaging of cancer cells. ACS Nano.

[8] Fujioka K, Hiruoka M, Sato K, Manabe N, Miyasaka R, Hanada S (2008). Luminescent passive-oxidized silicon quantum dots as biological staining labels and their cytotoxicity effects at high concentration. Nanotechnology.

[9] English DS, Pell LE, Yu Z, Barbara PF, Korgel BA (2002). Size tunable visible luminescence from individual organic monolayer stabilized silicon nanocrystal quantum dots. Nano Lett.

[10] Shiohara A, Prabakar S, Faramus A, Hsu CY, Lai PS, Northcote PT (2011). Sized controlled synthesis, purification, and cell studies with silicon quantum dots. Nanoscale.

[11] Kang Z, Liu Y, Tsang CHA, Ma DDD, Fan X, Wong NB (2009). Water-soluble silicon quantum dots with wavelength-tunable photoluminescence. Adv Mater.

[12] Holmes JD, Ziegler KJ, Doty RC, Pell LE, Johnston KP, Korgel BA (2001). Highly luminescent silicon nanocrystals with discrete optical transitions. J Am Chem Soc.

[13] Zou J, Baldwin RK, Pettigrew KA, Kauzlarich SM (2004). Solution synthesis of ultrastable luminescent siloxane-coated silicon nanoparticles. Nano Lett.

[14] Warner JH, Rubinsztein-Dunlop H, Tilley RD (2005). Surface morphology dependent photoluminescence from colloidal silicon nanocrystals. J Phys Chem B.

[15] Zhou Z, Brus L, Friesner R (2003). Electronic structure and luminescence of 1.1- and 1.4-nm silicon nanocrystals: oxide shell versus hydrogen passivation. Nano Lett.

[16] Puzder A, Williamson AJ, Grossman JC, Galli G (2003). Computational studies of the optical emission of silicon nanocrystals. J Am Chem Soc.

[17] Wilcoxon JP, Samara GA, Provencio PN (1999). Optical and electronic properties of Si nanoclusters synthesized in inverse micelles. Phys Rev B.

[18] Švrček V, Mariotti D, Kondo M (2009). Ambient-stable blue luminescent silicon nanocrystals prepared by nanosecond-pulsed laser ablation in water. Opt Express.

[19] Hua F, Erogbogbo F, Swihart MT, Ruckenstein E (2006). Organically capped silicon nanoparticles with blue photoluminescence prepared by hydrosilylation followed by oxidation. Langmuir.

[20] Warner JH, Hoshino A, Yamamoto K, Tilley RD (2005). Water-soluble photoluminescent silicon quantum dots. Angew Chem Int Ed.

[21] Kanemitsu Y, Futagi T, Matsumoto T, Mimura H (1994). Origin of the blue and red photoluminescence from oxidized porous silicon. Phys Rev B.

[22] Shiohara A, Hanada S, Prabakar S, Fujioka K, Lim TH, Yamamoto K (2010). Chemical reactions on surface molecules attached to silicon quantum dots. J Am Chem Soc.

[23] Warner JH, Tilley RD (2006). Synthesis of water-soluble photoluminescent germanium nanocrystals. Nanotechnology.

[24] Antipov A, Bell M, Yasar M, Mitin V, Scharmach W, Swihart M (2011). Luminescence of colloidal CdSe/ZnS nanoparticles: high sensitivity to solvent phase transitions. Nanoscale Res Lett.

[25] Pihlasalo S, Kirjavainen J, Hänninen P, Härmä H (2011). High sensitivity luminescence nanoparticle assay for the detection of protein aggregation. Anal Chem.

